# Preferred Strength of Noise Reduction for Normally Hearing and Hearing-Impaired Listeners

**DOI:** 10.1177/23312165231211437

**Published:** 2023-11-21

**Authors:** Rolph Houben, Ilja Reinten, Wouter A. Dreschler, Roland Mathijssen, Tjeerd M. H. Dijkstra

**Affiliations:** 1 Pento Audiological Centre, Amersfoort, The Netherlands; 2 Clinical and Experimental Audiology, Amsterdam UMC location AMC, Amsterdam, The Netherlands; 3 Embedded Systems Innovation, 2859 TNO, Eindhoven, The Netherlands; 4 Institute for Computing and Information Sciences, 6029 Radboud University Nijmegen, Nijmegen, The Netherlands; 5 Department of Women’s Health, University Clinic Tübingen, Tübingen, Germany; 6 Institute for Translational Bioinformatics, University Clinic Tübingen, Tübingen, Germany

**Keywords:** hearing aids, hearing loss, user preference analysis, subjective evaluation, paired comparison study

## Abstract

Preference for noise reduction (NR) strength differs between individuals. The purpose of this study was (1) to investigate whether hearing loss influences this preference, (2) to find the number of distinct settings required to classify participants in similar groups based on their preference for NR strength, and (3) to estimate the number of paired comparisons needed to predict to which preference group a participant belongs. A paired comparison paradigm was used in which participants listened to pairs of speech-in-noise stimuli processed by NR with 10 different strength settings. Participants indicated their preferred sound sample. The 30 participants were divided into three groups according to hearing status (normal hearing, mild hearing loss, and moderate hearing loss). The results showed that (1) participants with moderate hearing loss preferred stronger NR than participants with normal hearing; (2) cluster analysis based solely on the preference for NR strength showed that the data could be described well by dividing the participants into three preference clusters; (3) the appropriate cluster membership could be found with 15 paired comparisons. We conclude that on average, a higher hearing loss is related to a preference for stronger NR, at least for our NR algorithm and our participants. The results show that it might be possible to use a limited set of pre-set NR strengths that can be chosen clinically. For our NR one might use three settings: no NR, intermediate NR, and strong NR. Paired comparisons might be used to find the optimal one of the three settings.

## Introduction

Nowadays nearly all commercially available hearing aids contain a single-channel noise reduction (NR) algorithm. The goal of such an algorithm is to improve patient satisfaction when listening in a noisy background. NR algorithms in hearing aids have been shown to increase listening comfort at equal speech intelligibility (Chong & Jenstad, 2018). Commercially available hearing aids usually have the option of turning NR on or off or they contain a few (e.g., 3 or 5) factory default pre-sets that range from no NR, via intermediate to strong NR. Unfortunately, these default settings are mostly not documented well. NR algorithms are complex in that they depend on many parameters that can influence sound quality (e.g., maximum amount of gain reduction, the gain as a function of signal-to-noise ratio (SNR), noise tracking time constants, speech tracking time constants, and number of channels). Additionally, there are many different NR implementations (Hoetink et al., 2009) and hearing aid manufacturers have their own (scarcely documented) implementations that vary over their different product lines. Since specific prescription rules are not (yet) available, it is not known which NR setting is appropriate for an individual patient. Furthermore, it is not known how many distinct options of a certain NR parameter a device should offer in order to meet the needs of the individual listeners.

Preference for NR settings varies between individuals (Brons, Houben et al., 2014; Kubiak et al., 2022; Nelson et al., 2018; Völker et al., 2018; Wong et al., 2018). It is not clear how hearing impairment influences the preference for NR, as previous research showed inconsistent results. For instance, Kim et al. (2015) and Luts et al. (2010) did not find an effect of hearing impairment on preference for signals processed by NR. On the other hand, Sang et al. (2015) did find a difference in preference ratings for NR between normal hearing (NH) and hearing-impaired (HI) participants. In their paired comparison set-up, both NH and HI participants preferred NR over no NR, but the HI participants gave a better rating for the stimuli with active NR than the NH participants. The authors concluded that the lower preference score for NH listeners occurred because they are more affected by distortion caused by NR.

The studies described above all investigated differences in preference for NR turned on or off between NH and HI listeners. They did not investigate if NH and HI listeners differ in which setting of the NR algorithm was preferred. Neher (2014) used a coherence-based binaural NR algorithm to study preferences for NR strength. He found that strong NR (maximum attenuation was 30 dB) was more preferred by HI listeners with larger pure-tone average (PTA) than by listeners with a smaller PTA. In contrast, Arehart et al. (2015) concluded that the degree of hearing loss was not a significant factor in explaining the differences in quality ratings for different attenuation values of a binary-mask noise suppression technique. Brons, Dreschler et al. (2014) also found no significant difference between NH and HI listeners in the NR strength that they preferred. However, they did find that detection thresholds for distortion caused by NR were higher for HI participants than for NH participants. This higher detection threshold for distortions did not carry over to a significant difference in preference for NR strength. The authors’ interpretation was that HI listeners seem to tolerate fewer audible distortions than NH listeners do.

In a previous paper, different settings of a single parameter (the maximum gain reduction) were compared to investigate the preferences of NH listeners for NR strength in a laboratory experiment (Houben et al., 2011a). That work showed that, for the NR algorithm used, NH participants differed in their preferences for NR strength, if measured with enough repeats. This finding suggests that there is a relevant individual component of preferences for NR settings, although several repeats in an in-situ laboratory study might be required to reveal this. Subsequent research seems to confirm the finding that there are stable personal preferences for NR settings (Kubiak et al., 2022). However, it remains unclear if the amount of hearing loss is a determining factor for preferred NR strength.

Here we focus on NR strength, defined as the maximum gain reduction of an NR algorithm. Based on the literature we hypothesized that both NH and HI participants would have a large spread in preferences for NR strength. We further hypothesized that groups of NH and HI participants would differ in their preferred strength of NR. The direction of the preference difference is unknown. On the one hand, HI listeners might be less sensitive to signal distortions (Brons, Dreschler et al., 2014), and might thus accept stronger NR due to a larger positive effect of reduced noise. On the other hand, once signal distortions are audible, HI listeners might be less resilient to those distortions because the hearing loss itself can be regarded as a cause of signal distortion (interpretation of Brons, Dreschler et al., 2014).

Using hearing aid-based NR, we investigated if preferences for NR strength differ between NH and HI listeners for speech in babble noise. To achieve good sensitivity we used a paired comparison design and analyzed the data with a statistical model that was specifically developed for this task by Houben et al. (2011a). This model, the quadratic utility logistic (QUL) model, is described in more detail below. We also explored the range of preferences for NR strength between individuals, irrespective of hearing status. By using cluster analysis of the paired comparisons data, we aimed to find the optimal number of distinct NR strength settings for groups of participants with similar individual preferences. Finally, we estimated the number of paired comparisons required to adequately predict an individual's preference for NR strength. This was intended to provide valuable information for fine-tuning NR algorithms in clinical practice.

The following research questions were formulated:

*Research Question 1:* Do preferences for NR strength differ between NH and HI listeners?
*Research Question 2:* How many distinct settings are required to classify participants into similar groups based on their preferences for NR strength?
*Research Question 3:* How many paired comparisons are required to find the preferred NR strength for an individual?

## Methods

### Participants

Approval by the Medical Ethical Committee of AMC was obtained on 24 April 2008 (MEC 08/082). Participants were recruited from the patients of the Audiological Centre of the AMC and had to sign an informed consent prior to participation.

There were three groups of 10 participants in the study. The audiometric data are given in Appendix A. Two additional participants quit the experiment early stating that they did not hear any difference between the stimuli. Although their incomplete data sets were not used for analysis, their audiometric data are included in Appendix A for completeness.

The included number of participants was based on a power calculation with data from NH listeners from a previous study (Houben et al., 2011a). In that study, mean preference for NR strength was 7.5 dB with standard deviation of ±1.8 dB. If we assume the same measurement variance for our groups of participants, at least eight participants are required in each participant group to detect a difference in preference of 2.5 dB (between-participant design α = 0.05, β = 80, two-sided).

The audiometric inclusion criteria for the three hearing loss categories were as follows:

- NH: all hearing thresholds (octave frequencies ranging from 0.25 to 8 kHz) equal to or better than 20 dB hearing loss (HL) for both ears.
- Mild to moderate HI (HI-mild): hearing loss exceeding that of NH while the PTA hearing loss at 1, 2, 4 kHz (PTA_1, 2, 4 kHz_) of at least one ear was equal to or better than 40 dB HL.
- Moderate to profound HI (HI-moderate): hearing loss exceeding HI-mild (i.e., PTA_1, 2, 4 kHz_ of both ears worse than 40 dB HL.

The asymmetry of the hearing loss was small, see Appendix A. The mean value of the maximum asymmetry (=maximum difference between left and right air conduction threshold at each frequency from 250 to 8000 Hz) was 16 dB with a standard deviation of 10 dB. The mean PTA_1, 2, 4 Hz_ difference between the ears was 6 dB HL with a standard deviation of 6 dB HL. Hearing loss of the participants was sensorineural, there were no significant conductive losses.

The speech reception threshold (SRT) for consonant–vowel–consonant (CVC) words in quiet was also measured. The SRT is the sound pressure level at which the participant correctly repeated 50% of the phonemes. For the first five NH participants, no SRT measurement is available due to a measurement mistake. The mean age (with standard deviation) of the three groups was 47 ± 12 years, 61 ± 15 years, and 67 ± 7.5 years, for NH, HI-mild, and HI-moderate, respectively.

### Stimuli

The unprocessed stimuli were recordings of four sentences spoken by a female, taken from the VU-98 sentence materials (Versfeld et al., 2000). During the development of this Dutch speech-in-noise test, the sentences were optimized to have near-equal intelligibility in stationary noise (Versfeld et al., 2000). The sentences are, however, not homogenized for perceived listening comfort. Therefore, from these materials, four sentences (#49, 52, 58, 63) were selected to minimize possible differences in subjective preferences between different sentences. Sentence selection was done based on perceived listening comfort. The sentences were embedded in babble noise of which the long-term spectrum was matched to that of speech of the sentence materials. The sentences were rated for perceived listening comfort by three expert listeners. Perceived listening comfort was used rather than sound quality to avoid the (explicit or implicit) reference that plays a role when judging sound quality. Perceived listening comfort was rated on a 5-point scale ranging from *not comfortable* to *very comfortable*. The four sentences were selected to have equal scores and average listening comfort.

NR processing was done using a low-latency single-microphone NR algorithm (SNRA), implemented in Matlab. This algorithm has been used before to measure individual preferences for the strength of NR (Houben et al., 2011a, 2011b, 2013). The algorithm uses modulation-based spectral subtraction and has low complexity and low latency (Appleby & Groth, 2011; Groth & Nelson, 2005; Kates, 2017; Rosenstrauch, 2011). A detailed description of the algorithm can be found in Houben et al. (2011a, 2011b, 2013). Here, the algorithm is briefly described.

The incoming speech signal is first split into a signal and an analysis path. For the signal path, all filtering and processing is done in the time domain, and for the analysis path, this is done in the frequency domain (Groth & Nelson, 2005). The two paths are each analyzed with a 17-band frequency-warped filter bank (Kates & Arehart, 2005). The advantage of a frequency-warped filter bank over a conventional filter bank is that it has a non-uniform frequency representation very close to that of the auditory system.

The SNR is estimated for each frequency band using the estimation of the noise and speech signal (Rosenstrauch, 2011). During intervals in which no speech is detected, the input signal is used to calculate a noise estimate. The estimation of the speech signal is based on spectral and temporal characteristics of speech, using samples of the input signal of about 1 ms. The gain is calculated in the analysis path using the estimated SNR and Wiener optimal filtering theory (Smith, 2002), with a threshold for gain depth (=G_min_). This threshold was used to limit the strength of the NR. The calculated gain was subsequently applied to the signal in the signal path. The variable under investigation, the threshold for gain depth (G_min_), limits the maximum gain reduction that is applied by the SNRA to G_min_. Higher values of G_min_ led to more gain reduction. The variable G_min_ can be applied independently from the NR algorithm since it does not alter the gain function but only applies a threshold of maximum gain reduction. In the literature on spectral subtraction, this limit is also known as “spectral floor” (Berouti et al., 1979; Loizou, 2007).

**Figure 1.**
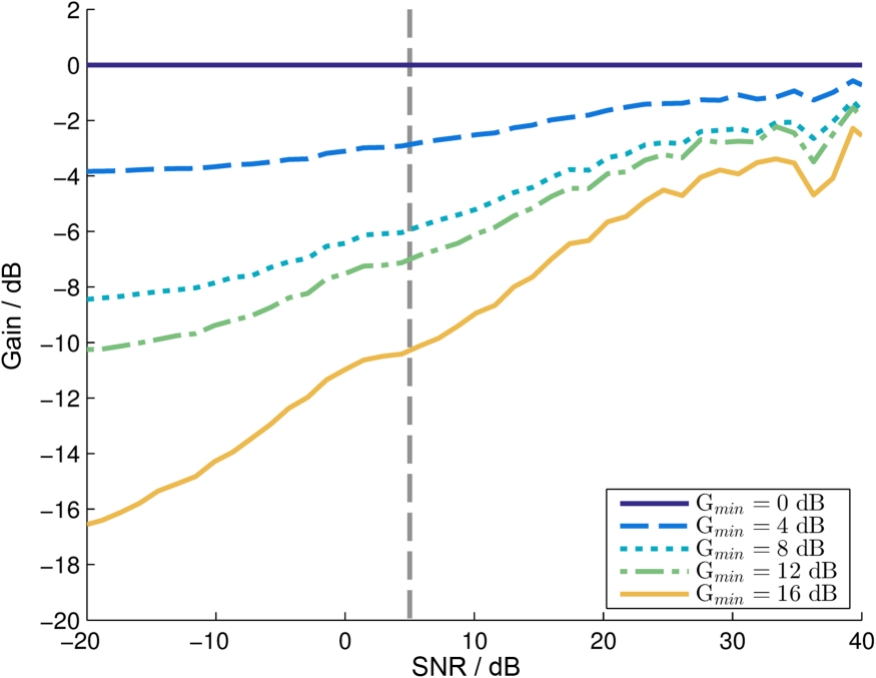
Calculated gain function for some values of G_min_ used. The gain was calculated by comparing the processed and unprocessed sound signals for one sound file which consists of all four sentences concatenated. The striped vertical line at +5 dB indicates the long-term average SNR of the stimuli.

Figure 1 shows the estimated realized gain of our algorithm as function of the estimated input SNR. The processed sentences were analyzed based on the four sentences that were chosen for this experiment. These sentences were placed one after another and processed by the NR algorithm in loops such that at least 60 s of the processed output file could be removed to stabilize the algorithm. The gain that was applied by the algorithm was calculated by comparing the processed (output) signal to the input signal for separate time-frequency bins (window length was 512 samples, with a sample frequency of 16 kHz). Note that due to estimation errors, the maximum gain in this plot can be larger than G_min_.

By removing the estimated noise, the loudness of the speech-in-noise signal is inevitably affected. To correct for this, NR algorithms commonly apply a correction that restores the overall gain. Our algorithm restores the gain by matching the Root mean square (RMS) of the output signal to that of the input. The correction is thus done on the overall speech-in-noise signal.

### Experimental Design

At the start of the visit, hearing status was checked for each participant by means of pure-tone audiometry. Following audiometry, they participated in a paired comparison listening test which is explained in the next two paragraphs.

A between-participants design was used, the three hearing loss groups each containing 10 participants. The main experimental parameter was G_min_, which is explained in the previous section. Ten values of G_min_ were used (0, 4, 6, 7, 8, 9, 10, 12, 16, 18 dB), each of which was compared to all the others, leading to 10 × 9 unique comparisons (including AB, BA to prevent bias in the presenting order). Each comparison was done twice (test and retest, without a pause in between) leading to 180 paired comparisons per participant. This value was a trade-off between measurement accuracy (more runs are better) and total time required to do the experiment (less is better). On average, a session took about 1.5 h.

In each trial, participants had to listen to two successive processed speech-in-noise stimuli and choose which they preferred. The participants were asked the following question “Imagine that you will have to listen to these signals all day. Which sound would you prefer for prolonged listening?” The question was intentionally stated in a broad context. The reason is that we were primarily interested in general preference and not in perceived specific signal qualities such as “speech quality” or “amount of background noise.” Before commencing with the paired comparisons listening test, a training session was performed with 20 paired comparisons with similar stimuli. The data obtained during the training session were discarded.

The experimental set-up consisted of a Focusrite Saffire Pro 10 audio interface and a Presonus HP40 headphone buffer with Sennheiser HDA200 headphones. All stimuli were presented bilaterally with an input SNR of +5 dB. This value was chosen because it is representative of real-life situations, which is shown in a field study by Wu et al. (2018) who found that most realistic SNRs were between 2 and 14 dB. We chose a lower value of this range such that it is sufficiently challenging for both the listener and the NR algorithm and in line with research by Houben et al. (2013). The nominal speech level was 70 dB Sound pressure level (SPL), and the noise level was 65 dB SPL. For the participants in the HI-mild and HI-moderate groups, the sound signals were amplified according to the National Acoustics Laboratories-Revised Profound (NAL-RP) fitting rule (Dillon, 2001). The sound signals for the left and right ear received the same NAL-RP amplification, based on the ear with the smallest hearing loss. This approach was possible because the asymmetry in the hearing loss was small; see Appendix A.

### Statistical Analysis

#### The QUL Statistical Model

The dichotomous paired comparison data were analyzed with the QUL model developed by Houben et al. (2011a, 2011b) for NR preference. Briefly, the model is based on the assumption of a trade-off between speech distortion caused by the NR algorithm and the amount of residual background noise. The model takes this trade-off into account for individual listeners by combining a quadratic utility model with Logistic regression. The QUL model estimates the value of G_min_ that corresponds to the participant's highest preference ( $G_{\min}^{\mathrm{opt}}$ ). To avoid extrapolation, values of $G_{\min}^{\mathrm{opt}}$ were restricted to the possible range of 0–18 dB.

#### Response Feature Analysis

The QUL model was applied to the data for each individual participant. The obtained individual optimal values were subsequently analyzed to investigate possible differences between the three participant groups. This approach is known as response feature analysis (Dupont, 2009). With response feature analysis, multiple individual responses are reduced to features that capture the attribute of interest. Subsequently, this response measure was analyzed in a fixed-effects one-way analysis. Response feature analysis has the advantage that the results are clearly interpretable. Another advantage is that the use of a single value per individual (i.e., $G_{\min}^{\mathrm{opt}}$ ) does not require estimation of the correlation structure of multiple observations that would be required with a more elaborate model. A disadvantage of response feature analysis is that information in the correlation between the repeated measures of individual participants is not used. This may lead to some loss of statistical power.

The QUL model was implemented in R and the one-way between-participants analysis was done in Matlab with the standard Kolmogorov–Smirnov and Kruskal–Wallis tests. The correlation between the preferred individual G_min_ levels and hearing loss was calculated using Spearman correlation (standard Matlab function `corr`). Hierarchical cluster analysis was done with R function “hclust” using the Ward criterion (method = “ward.D”). The distances between win counts were calculated using R function “dist” using the Manhattan (L1) distance metric (method = “manhattan”). We chose hierarchical clustering over alternatives like *k*-means clustering, as this hierarchical clustering gives insights into clustering quality for different numbers of clusters. We chose Ward's minimum within-cluster variance as method, as this method “tends to find same-size, spherical clusters” (Everitt et al., 2011). Because we wanted to divide the participants into roughly equal groups, Ward's method was the best choice. However, Ward's method is sensitive to outliers thus we used the Manhattan (L1) distance as metric, as this distance is more robust against outliers.

## Results

**Figure 2.**
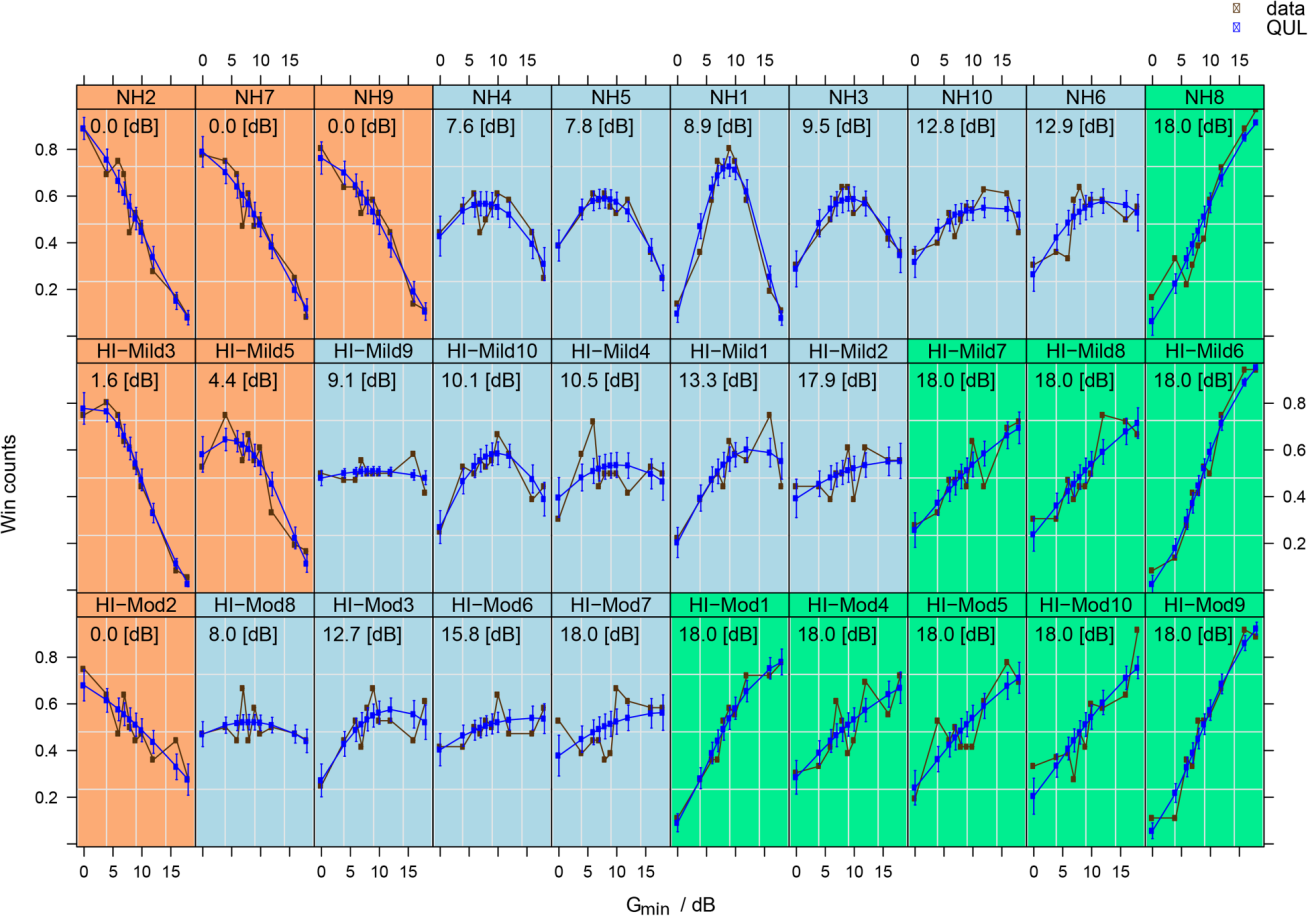
Win counts (as fraction of the total) and model fits obtained from the QUL model for each participant. Win counts are shown in brown and are connected by brown line segments for clarity. QUL fits are shown in blue. The first row shows data for the NH participants, the second row for the HI-mild participants, and the bottom row for the HI-moderate participants. The participants in each row are ordered by increasing value of $G_{\min}^{\mathrm{opt}}$ . The background color indicates membership in the cluster analysis; see the section “Data-driven analysis of preference”. The calculated value of $G_{\min}^{\mathrm{opt}}$ (in dB) is also given (text below the participant designation).

Paired comparison data can be displayed graphically by expressing the results as win counts: the number of times each G_min_ was chosen over its alternatives. Figure 2 shows the win counts for each participant. Figure 2 also shows the fits from the QUL model (blue curves). The model fits represent the preference of the participant as a function of NR strength. The error bars in the model fits show the pointwise standard errors in the model predictions for the G_min_ levels used. Note that the model shows the trends for the vast majority of listeners, although there are occasional exceptions, like participant HI-mild4. The calculated preferred value of G_min_ for each participant ( $G_{\min}^{\mathrm{opt}}$ ) is the value of G_min_ that corresponds to the highest preference.

### Response Feature Analysis

**Figure 3.**
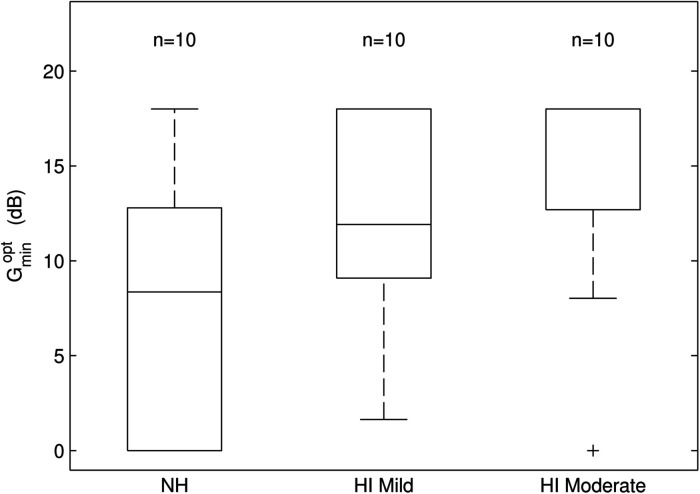
Box plot of $G_{\min}^{\mathrm{opt}}$ for each hearing loss category (NH, HI-mild, HI-moderate). The median is indicated by a horizontal line and was 8.2 dB for NH, 11.6 dB for HI-mild, and 15.7 dB for HI-moderate. The mean ranks of $G_{\min}^{\mathrm{opt}}$ were significantly different between NH and HI-moderate (*p* < .05; see text).

The first research question was to find out whether preference for NR strength differs between NH and HI listeners. To answer this, a response feature analysis of the data of the three participant groups was performed. Figure 3 shows box plots of $G_{\min}^{\mathrm{opt}}$ for each of the three hearing loss categories.

Because the analysis of variance (ANOVA) assumption of normality of the residuals was not met (a Kolmogorov–Smirnov test gave *p* < .001), we analyzed the data with a non-parametric Kruskal–Wallis test with hearing loss category as the main effect. Prior to this analysis, we checked for homoscedasticity with Bartlet's test for equal variances. The effect was not significant (Bartlett's statistic = 0.06; *p* = .97), indicating that the variances were not different for the three hearing loss categories, and that a Kruskal–Wallis test could be used.

The Kruskal–Wallis test gave a significant effect of hearing loss (χ^2 ^= 6.2; *p* < .05). Post hoc testing (Bonferroni corrected with α = 0.05 and *n* = 3) showed that the mean ranks of the NH and HI-moderate groups differed significantly (*p* < .05).

### Correlation Between Hearing Loss and Age

**Figure 4.**
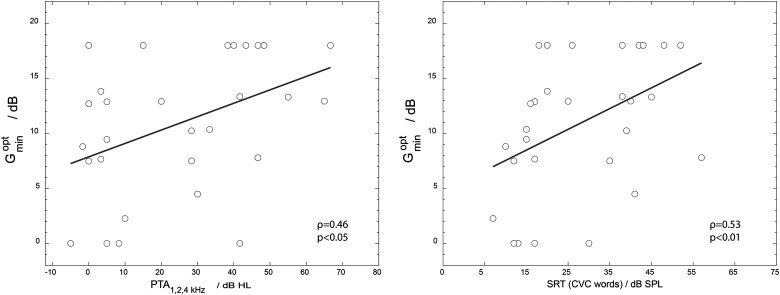
Scatter plot of $G_{\min}^{\mathrm{opt}}$ values against PTA_1, 2, 4 kHz_ (left panel) and SRT (50% correct CVC words in quiet; right panel). The test ear was the ear with the lowest PTA_1, 2, 4 kHz_. A trend line is shown in the figure which is the result of a standard linear regression.

To investigate the effect of hearing loss on the preference for NR strength, Figure 4 (left panel) shows a scatterplot of $G_{\min}^{\mathrm{opt}}$ against the PTA_1, 2, 4 kHz_ for the better ear. The better ear was defined as the ear with the lowest PTA_1, 2, 4 kHz_.

Because a Kolmogorov–Smirnov test showed that both PTA_1, 2, 4 kHz_ and $G_{\min}^{\mathrm{opt}}$ were not normally distributed *p* < .001, the non-parametric Spearman correlation was used. The correlation was moderate: ρ = 0.46; *p* < .05. The Spearman correlation between $G_{\min}^{\mathrm{opt}}$ and the SRT for CVC words in quiet was also moderate: ρ = 0.53, *p* < .01.

**Figure 5.**
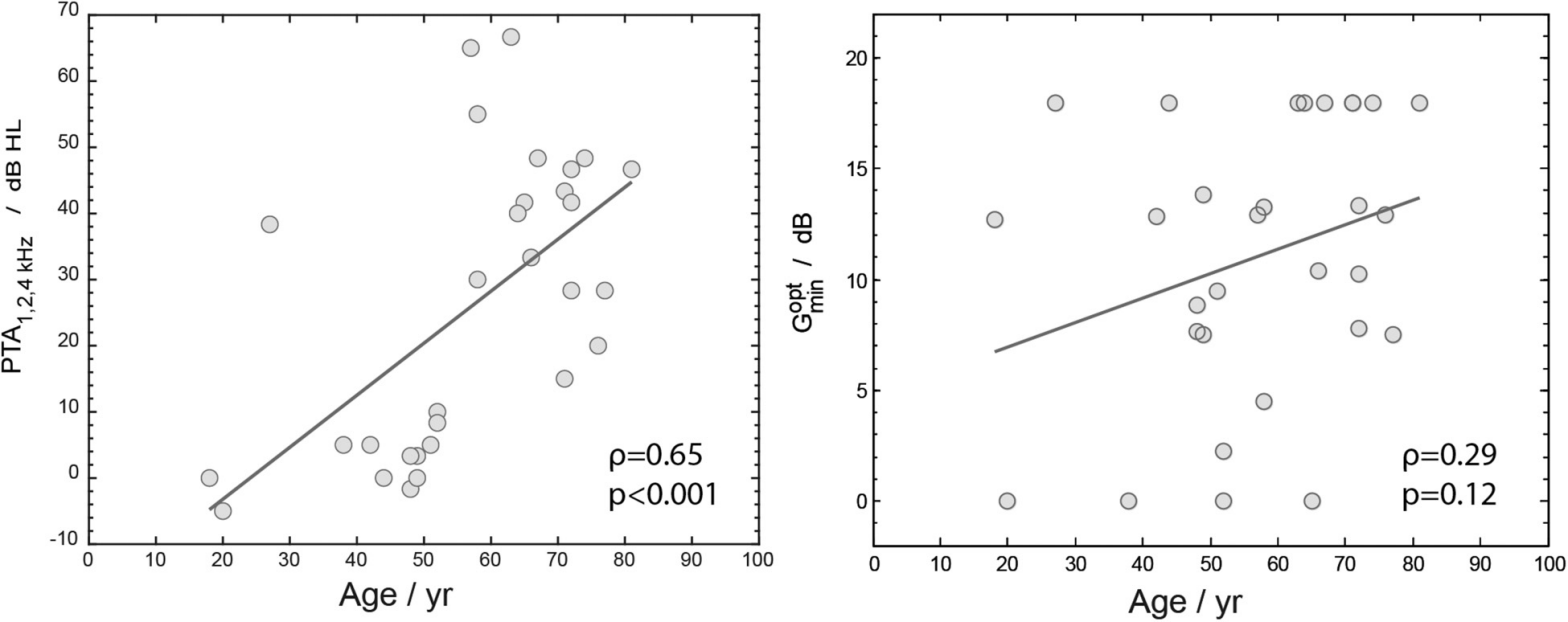
The left panel shows a scatter plot of PTA_1, 2, 4 kHz_ against age. The right panel shows a scatter plot of $G_{\min}^{\mathrm{opt}}$ against age. A trend line is shown in the figure which is the result of a standard linear regression.

We investigated the relation between age and hearing loss, and between age and $G_{\min}^{\mathrm{opt}}$ to find out whether the preference results are biased due to age, see Figure 5. A one-way ANOVA on age with hearing loss category as predictor showed that the hearing loss groups differed in age: *F*(2, 29) = 13.31; *p* < .001. The PTA_1, 2, 4 kHz_ was significantly correlated with age: ρ = 0.65, *p* < .001 (Spearman correlation), but $G_{\min}^{\mathrm{opt}}$ was not: ρ = 0.29, *p* = .12 (Spearman correlation).

### Data-Driven Analysis of Preference

**Figure 6.**
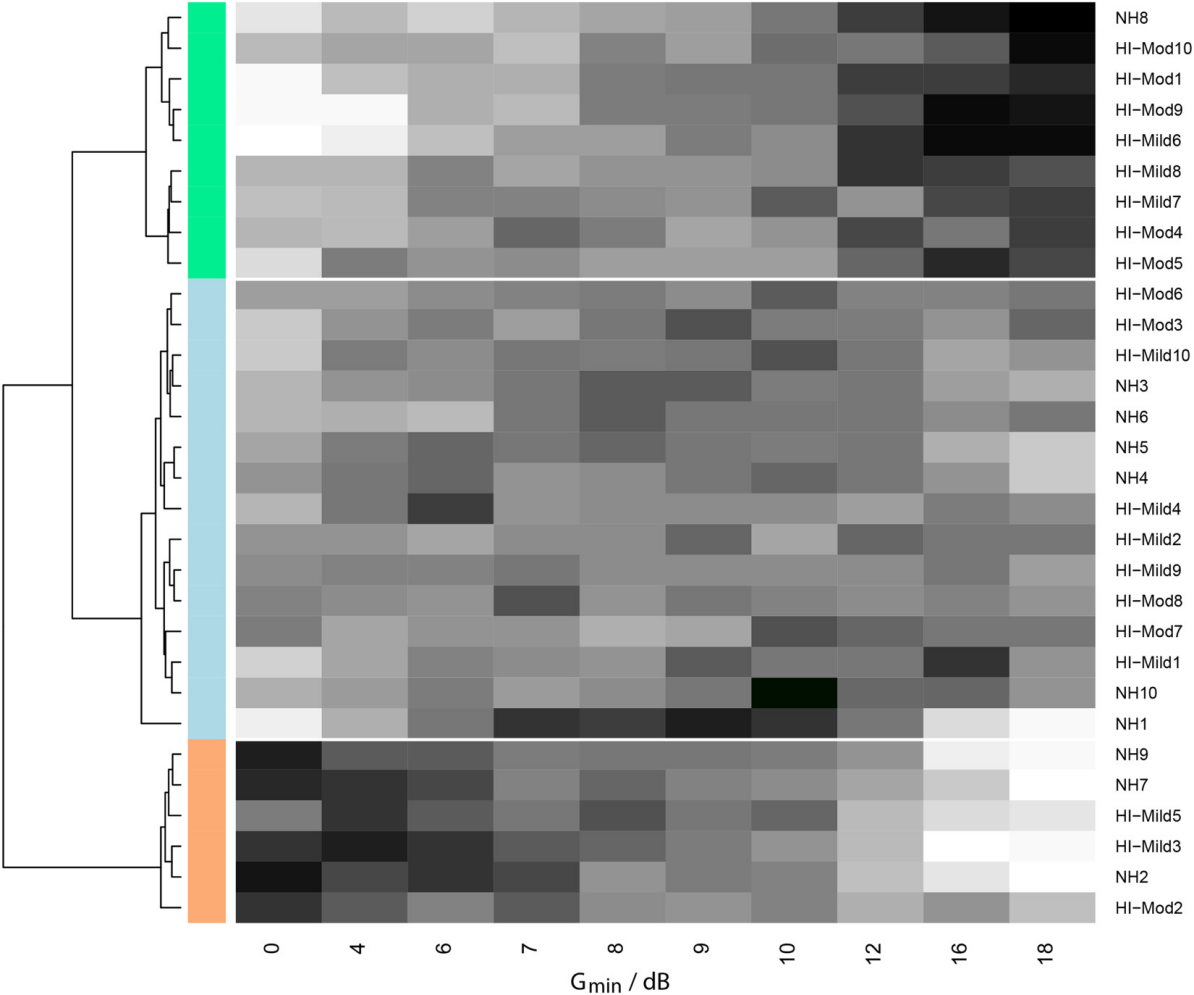
Hierarchical cluster analysis of the win-count data. The different gray scales represent the win counts (white is low, black is high).

The second research question was to define the required number of distinct NR settings to be able to classify our participants into similar groups based on their NR preference. To answer this question a cluster analysis was performed. Figure 6 shows the results of the hierarchical cluster analysis The dendrogram is shown on the left. The horizontal distance on the dendrogram represents the calculated Manhattan (L1) distance between the merged clusters.

From left to right, the dendrogram shows the possible different levels with different numbers of clusters starting with two clusters in the first level and finishing with 30 separate clusters, one for each participant (see James et al., 2013 for a tutorial on hierarchical clustering and dendrograms). In the dendrogram, we can distinguish two stable clusterings: one with two clusters and one with three clusters. Stable clusterings are those that persist over a large horizontal range in the dendrogram. The clustering with two clusters has one group with six participants that prefer no NR and one group with 24 participants that prefer some non-zero level of NR. This clustering supports the choice that some hearing aid manufacturers make: NR is either on or off. The other stable clustering is one with three clusters. This clustering consists of the same group of six participants that prefer no NR and separates the remaining 24 participants into 9 and 15 participants that differ in the strength of NR they prefer. The next (unstable) clustering with five clusters does not provide us with additional insight. We focus on the clustering with three clustering for the rest of this manuscript. For each of these three clusters, the middle part of Figure 6 shows the win-count data for each participant. The different gray scales represent the win counts (white is low, black is high). Participant identity is indicated at the right of the figure. The clusters can be interpreted as one group that prefers zero NR (coded orange in Figure 6), one group that prefers an intermediate level of NR (light blue in Figure 6), and one group that prefers a high level of NR (green in Figure 6).

**Figure 7.**
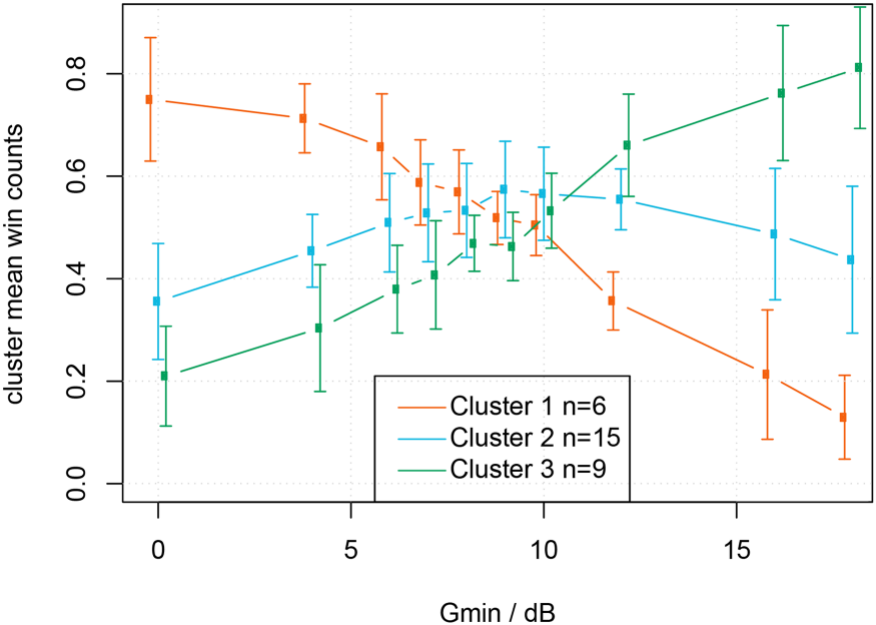
Cluster analysis: win count for each of the three clusters is calculated by averaging the win counts of each of the participants in a cluster. Error bars denote the standard deviation across participants.

The average win-count data for each of the identified relevant clusters with respect to G_min_ gives us information on the differences in preferences between the different clusters throughout the range of G_min_. Figure 7 shows the average win-count data (with standard deviation). Note that these data were derived directly from the participants’ answers, without information on the hearing loss or hearing loss group.

The preference in Cluster 1 (shown in orange) is for low G_min_ (with mean $G_{\min}^{\mathrm{opt}}$ = 0 dB), the preference in Cluster 2 (shown in blue) is for intermediate G_min_ (mean $G_{\min}^{\mathrm{opt}}$ = 10.8 dB), and the preference in Cluster 3 (shown in green) is for high G_min_ (mean $G_{\min}^{\mathrm{opt}}$ = 18 dB).

The last question addressed is how many paired comparisons are needed to place a participant in one of the three preference clusters. As 180 paired comparisons (the number we used in this study) is too many for clinical practice, only G_min_ levels of 0, 7, 8, 9, 10, and 18 dB were used in a simulation study. These G_min_ levels coincide approximately with the $G_{\min}^{\mathrm{opt}}$ from each of the three clusters. Response data were used from paired comparisons between G_min_ = 0 dB and each of G_min_ = 7, 8, 9, or 10 dB (eight pairs, as each could be the first or second of a pair), between G_min_ = 18 dB and each of G_min_ = 7, 8, 9, or 10 dB (eight pairs) and G_min_ = 0 dB and G_min_ = 18 dB (two pairs). To increase the weight of these last pairs, the data were used twice for a total of 8 + 8 + 2 × 2 = 20 pairs. The data contained only two pairs of comparisons between G_min_ = 0 dB and G_min_ = 18 dB and hence only two out of 18 pairs compare preference for Cluster 1 versus 3 (cluster numbering as in Figure 7). As this is an important comparison, we increased the weight of these comparisons by including them twice, increasing the fraction from 2/18 to 4/20. As there were two repetitions of all paired comparisons per participant, the total number of paired comparisons per participant was 40.

Subsequently, a resampling procedure was used on these paired comparisons, simulating a listening experiment where pairs were presented in random order. In resampling, each paired comparison is equally likely to be picked and each pair can be picked only once: uniform sampling without replacement. Note that this resampling is an average-case scenario: one could easily improve on this by adaptive sampling, where one uses previous responses to update the sampling strategy. For example, after a participant has indicated 6 times a preference for G_min_ = 0 dB over G_min_ = 18 dB one is unlikely to get new information by asking the participant a seventh or eighth time (as is done in the resampling procedure.

Getting back to the resampling procedure, 5–40 pairs were sampled (in Step 5) from the 40 paired comparisons selected previously and the Euclidian distance was calculated between the win counts of the sample and the mean win counts of each cluster as shown in Figure 7. Lastly, the cluster with the shortest Euclidean distance from the sample was picked. This simulation was run 500 times to estimate how often the sample ended up in the correct cluster (i.e., the cluster assigned to the participant based on all 180 paired comparisons).

**Figure 8.**
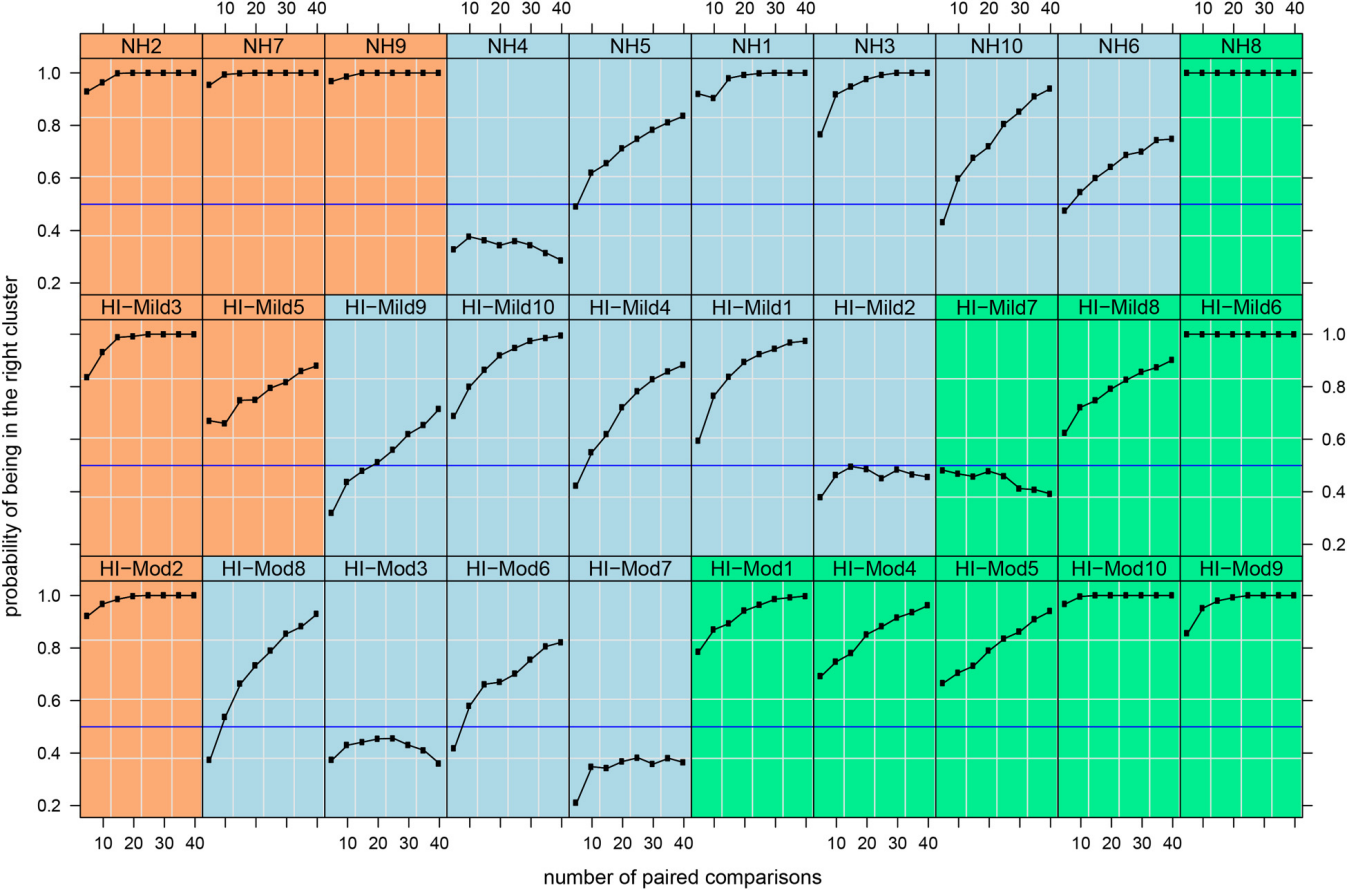
Probability of selecting the correct cluster (green, blue, or orange) for each participant based on a random subset of the paired-comparison data. Data shown represent resampling from paired comparisons between three distinct groups based on G_min_ (0 dB, 7, 8, 9, 10 dB, and 18 dB). The color coding represents the cluster the participants were assigned to by the cluster analysis (see Figure 5). The horizontal blue lines indicate a probability of 0.5.

The results of this simulation are shown in Figure 8. For nearly all participants in the first and third cluster, after 20 paired comparisons a probability of >80% of choosing the correct cluster was reached. In the middle cluster participants NH4, NH5, NH6, HI-mild2, HI-mild9, HI-mod3, HI-mod7, and HI-mod8 needed more paired comparisons to reach a probability of >80% or did not reach this percentage at all. Apparently, a significant fraction of participants in this cluster needs a wider range in G_min_ to adequately estimate the actual preference. This results in more comparisons than the G_min_ range in our simulation. We have no explanation for why the probability of choosing the correct cluster of HI-mild7, which belongs to the third cluster, was significantly lower in comparison to the other participants in this cluster.

## Discussion

### Q1: Does Preference for NR Strength Differ Between NH and HI Listeners?

In line with our hypothesis, there was a significant difference in mean preference between NH participants and HI participants with moderate hearing loss. On average, the participants with moderate hearing loss preferred stronger NR than the NH participants did. Also, there was a moderate positive correlation between PTA_1, 2, 4 kHz_ and $G_{\min}^{\mathrm{opt}}$ , and between SRT and $G_{\min}^{\mathrm{opt}}$ . There was no significant difference in average NR strength preference between NH participants and HI participants with mild hearing loss and between HI participants with mild or moderate hearing loss.

The effect of age was explored by calculating the Spearman correlation between age and $G_{\min}^{\mathrm{opt}}$ , and between age and PTA_1, 2, 4 kHz_. Age and PTA_1, 2, 4 kHz_ were significantly correlated. This was expected because age was not a selection criterion for participant inclusion and hearing loss generally increases with age (Hoffman et al., 2017). $G_{\min}^{\mathrm{opt}}$ was not significantly correlated with age, which suggests that the correlation between hearing loss and $G_{\min}^{\mathrm{opt}}$ was not primarily driven by age. These results imply that a person with more severe hearing loss is likely to prefer stronger NR, irrespective of age.

Houben et al. (2011b) studied preferences for NR strength in a similar experiment. The average value of $G_{\min}^{\mathrm{opt}}$ was only 0.3 dB larger for HI participants than for NH participants and this difference was not significant. They used three different background noises and two different NR algorithms. They tested 10 NH participants and 7 HI participants. The current study included more HI participants and divided them into groups that differed in hearing loss. Additionally, the current design included more NR strength levels which resulted in more pairwise comparisons for the single NR algorithm. These methodological differences may explain why the effect of hearing loss on NR strength preference was significant here whereas it was not in Houben et al.’s (2011b) study.

The direction of preference (those with more severe hearing loss are likely to prefer stronger NR) is in line with the hypothesis that HI listeners are less sensitive to signal distortions (Brons, Dreschler, et al., 2014). Neher and Wagener (2016) also found that HI listeners preferred stronger NR. The average $G_{\min}^{\mathrm{opt}}$ was 8.2 dB for the NH group, 11.6 dB for HI-mild, and 15.7 dB for HI-moderate. For the same NR algorithm and noise type, the average $G_{\min}^{\mathrm{opt}}$ was 4.3 dB in Houben et al. (2011b) for 17 participants (NH and HI participants combined). The cause of this difference is not clear. Perhaps the presence of different types of background noise in the previous study influenced the preferred NR strength.

We found a significant effect of hearing loss on preferred NR strength, although the effect size was only moderate (ρ = 0.46). This could be explained by the large individual differences in preferences (see Figure 3). Thus, there is substantial variation in preference irrespective of hearing status. Other individual traits might underlie this large spread of preferences for NR strength. Houben et al. (2011b) also found a large spread of preferences for NR strength for both NH participants and HI participants, with standard deviations of 3.6 dB and 4.0 dB, respectively. Several studies have investigated individual factors in relation to personal preferences for signal processing features in hearing aids (Neher & Wagener, 2016; Perry et al., 2019; Recker et al., 2020; Sugiyama et al., 2022). Unfortunately, for NR strength preference most of the investigated personal traits or factors were not predictive, including the acceptable noise level (ANL; Neher & Wagener, 2016; Recker et al., 2020), self-reported sound personality traits (Neher & Wagener, 2016), and the detection threshold for signal distortions (Brons, Dreschler et al., 2014; Neher & Wagener, 2016). On the other hand, Neher (2014) cautiously concluded that lower working memory might be related to preference for stronger NR. Individual preferences for NR settings, and thus NR strength, are complex and not easily predicted by subjective or objective measures.

A possible and promising explanation for the spread of preference is the individual trade-off between noise tolerance and distortion tolerance. Several researchers have used this trade-off theory to explain individual differences in preferences for NR settings (Brons, Houben, et al., 2014; Houben et al., 2013; Luts et al., 2010; Neher & Wagener, 2016; Reinten et al., 2019; Rohdenburg et al., 2005; Völker et al., 2018). Sugiyama et al. (2022) assessed individual differences in tolerance for signal distortion and residual noise using a single-channel speech enhancement algorithm. They found that their participants (*N* = 32) could be divided into two equal groups: those who are sensitive to distortions and those who are sensitive to noise. Kubiak et al. (2022) also found stable responses for participants (*N* = 30) who were classified either as “noise-haters” or “distortion-haters” in complex listening situations with different maskers. In the results shown in Figure 2, there are some participants of whom we can expect an individual trade-off to be made. For instance, the preference curve of NH1 peaks in the middle of our range of NR strengths suggesting this participant prefers an equal balance between speech distortion and noise attenuation. The preference curve of NH8 strongly suggests a preference for as much noise attenuation as possible in spite of the inevitable speech distortions, and vice versa for NH2. HI-mild9, however, which shares a similar $G_{\min}^{\mathrm{opt}}$ as NH1, does not seem to show such a clear trade-off in preference as the preference curve is much flatter. Perhaps this participant is more indifferent in preferring a certain NR strength and is bothered less by noise as well as distortion effects. In future research, we aim to study this individual trade-off for noise and distortion tolerance in NR strength preferences.

### Q2: How Many Distinct Settings Are Required to Classify Participants Into Similar Groups of NR Strength Preference?

The results of the cluster analysis showed that for our NR algorithm, the participants could be divided into three groups with similar preferences. The $G_{\min}^{\mathrm{opt}}$ values for the three clusters were 0 dB (no NR), 10.8 dB, and 18 dB; see Figure 6. Figure 7 shows the mean win counts for each of the three clusters. This figure clearly illustrates the differences in individual preference which can be categorized as no NR, medium NR, and strong NR. The results imply that it seems possible to limit the number of choices of NR strengths for the clinician. Specifically, for a NR algorithm similar to the one used in this study and with a similar range of NR strengths, three levels of NR strength might suffice. We do not want to imply, however, that the three settings of NR strengths suffice in all other NR algorithms, which can differ in signal processing strategies as well as in the range of NR strengths. For instance, should we have included even a stronger threshold for G_min,_ we might have concluded that four settings were required to be able to accommodate all preferences for NR strength. The results also imply that we can assume that simply offering an NR-on or NR-off option in a hearing aid is too limited, as there are listeners who do not prefer the maximum, nor do they prefer the minimal amount of gain reduction. The actual amount of three clusters is only a starting point based on a limited data set.

In the results of the cluster analysis, we can see that there was a trend for those with more severe hearing loss to belong to a cluster with a higher preferred NR strength, which is in line with our results for Q1. Most NH participants fell in Cluster 1 (low NR strength) or 2 (intermediate NR strength). HI-mild and HI-moderate participants fell more in Clusters 2 or 3 (high strength) than in Cluster 1. This suggests that most HI participants should be fitted with a device with NR active. However, the categorization of a participant in a cluster was not clear-cut because all three clusters contained one or more participants for the HI-moderate group. So there are, at least some, moderately HI participants who prefer no NR. These results reinforce earlier findings that the amount of hearing loss cannot reliably predict the preference for NR strength for an individual (Arehart et al., 2015; Brons, Dreschler et al., 2014). Therefore, instead of selecting the NR strength based on the audiogram, one could attempt to measure the preference with a short preference measurement.

### Q3: How Many Paired Comparisons Are Required to Find the Optimal Setting for an Individual?

If one could a priori categorize a participant into one of the three clusters in a reliable way, one could optimize the NR for that individual. Paired comparisons have been used for many decades to evaluate preferences for hearing aid features (Byrne, 1994; Neuman et al., 1995; Zerlin, 1962), and are often used for measurement of user preference for NR settings (Brons, Houben, et al., 2014; Marzinzik & Kollmeier, 2003; Smeds et al., 2010). In this study, we have used a complete set of two alternative forced-choice paired comparisons analyzed with the QUL model. There are other methods of paired comparison testing such as offering a “tie” option or offering more alternatives in one comparison, as are there other statistical methods and models (i.e., the Bradley–Terry–Luce model or the Elimination by Aspects model) to analyze paired comparison data (Cattelan, 2012; Tsukida & Gupta, 2011). In clinical practice, however, paired comparisons are not routinely used for hearing aid fitting and fine-tuning (Amlani & Schafer, 2009). In a survey of 251 audiologists, Anderson et al. (2018) found that the vast majority of the respondents used default settings of the manufacturer (58%) or their own expertise (38%) for fitting an NR feature in a hearing aid. An understandable reason for not using paired comparisons is that they take considerable time, which is not feasible in clinical practice. Therefore, we investigated whether the optimal level of NR strength can be found with a small subset of paired comparisons.

Figure 8 shows for each participant the probability of choosing the correct cluster against the number of paired comparisons. For five participants (e.g., NH4), the probability did not reach 50%. This implies that for these participants a higher probability, if possible, would require more comparisons, or a wider range of included NR strengths. The majority of these participants are in the middle cluster. However, for most of the participants in the first and third clusters, the probability of choosing the correct cluster was close to 100% after approximately 10–20 paired comparisons. Such a measurement should take about 5–10 min. For a clinical setting, we suggest using a limited set (e.g., 15 comparisons, or only the comparisons of the $G_{min}^{opt}$ values of the three clusters). If the listener cannot complete the task or if the result is inconclusive (i.e., no $G_{min}^{opt}$ can be found using the QUL model), the middle setting should be selected. A shortened preference measurement should be applicable for many hearing aid users in clinical practice, or alternatively could be incorporated in a hearing aid using machine learning, as suggested by Søgaard Jensen et al. (2019).

Although the NR algorithm used in this study is comparable to NR systems used in modern hearing aids, the optimal NR strength levels from this experiment apply only to the limited conditions tested in this study. Moreover, this study did not account for the effects of other non-linear processing, such as amplitude compression. We do not know if preferences for NR strengths differ at other input SNRs. However, since the chosen input SNR of +5 dB is representative of real-life scenario this limitation does not influence the conclusions of this work.

It is important to discuss other possible effects of our signal processing that might contribute to the differences in preference for NR strength. It is currently not known if or how the chosen hearing aid fitting rule (i.e., the amplification strategy to compensate for hearing loss) influences the preference for NR. To achieve results that are relevant to H users, one needs to use fitting rules. Fitting rules are used in hearing aids to prescribe individual, frequency-dependent amplification to compensate for the personal hearing loss. Research on preference with HI participants is thus complicated by the individual frequency-dependent hearing loss. The combination of the hearing loss with the chosen frequency-dependent fitting rule will determine the amount of spectral coloring relative to participants without hearing loss. Note that even without the use of frequency-dependent amplification there is some spectral coloring relative to NH (e.g., high frequencies can be attenuated by age-related hearing loss). We chose to apply the linear NAL-RP filter because it resembles the commonly used NAL fitting rule and it avoids the known complicated interactions with non-linear amplification (Brons et al., 2015). To what extent our choice of fitting rule (NAL-RP) has influenced the preference results is unknown. More specifically it is known that listeners might prefer less gain than prescribed by the NAL-RP fitting rule (e.g., Humes et al., 2000, 2001). This “too harsh” sound might have influenced the preference. However, due to the RMS correction that effect seems limited because overall the NR acts on all frequencies (the noise was spectrally matched to the speech).

## Conclusions

The results showed that preferred NR strength in hearing aids was moderately correlated with the degree of hearing loss. An individual with more severe hearing loss is likely to prefer stronger NR. However, there was large variation in preference for NR strength. Therefore, choosing NR strength based on the audiogram alone can result in suboptimal hearing aid fitting. For the conditions tested in this study, three distinct settings of NR strength sufficed to adequately accommodate individual preference. Thus it might be possible to use a limited set of pre-set NR strengths that can be chosen clinically. For most participants, the appropriate setting could be found with about 15 paired comparisons. For clinical practice, we advise using hearing status as a (first) guess to select the NR strength and then measuring individual preferences by using a limited number of paired comparisons.

## Appendix A

Age, air conduction thresholds (dB HL) for each frequency and ear, PTA_1, 2, 4 kHz_ and SRT values for all participants.

**Table:**

	Participant	Age (years)	Air conduction threshold (dB HL)													SRT
			AD						AS						Best ear	(50% CVC correct in quiet)
			250	500	1	2	4	8	250	500	1	2	4	8	PTA_1, 2, 4 kHz_	
			Hz	Hz	kHz	kHz	kHz	kHz	Hz	Hz	kHz	kHz	kHz	kHz	dB HL	dB SPL
Normal hearing																
	NH1	48	0	0	0	−5	0	0	0	0	0	−5	0	5	−2	—
	NH2	38	5	5	5	5	5	15	20	15	10	5	10	20	5	—
	NH3	51	10	5	5	5	10	5	5	5	5	5	5	0	5	—
	NH4	49	0	5	0	0	0	10	5	5	0	0	0	0	0	—
	NH5	48	5	5	10	5	10	10	15	10	5	0	5	15	3	—
	NH6	42	20	15	5	5	5	15	15	10	5	5	10	15	5	27
	NH7	52	0	5	10	5	15	10	5	10	15	15	35	15	10	27
	NH8	44	0	0	5	5	5	5	0	0	0	0	0	5	0	28
	NH9	20	5	5	5	0	−5	−5	0	5	−5	−5	−5	−5	−5	22
	NH10	18	5	5	0	5	−5	5	5	5	0	5	−5	−5	0	26
HI mild																
	HI-mild1	49	15	15	5	0	5	30	15	10	5	0	5	30	3	30
	HI-mild2	76	20	10	5	15	40	80	10	0	10	25	70	95	20	35
	HI-mild3	52	0	5	10	5	15	10	5	10	15	15	35	15	10	17
	HI-mild4	66	65	45	25	15	60	90	70	55	35	50	85	85	33	25
	HI-mild5	58	10	5	15	35	55	80	10	10	20	30	40	45	30	51
	HI-mild6	71	25	20	15	15	15	60	25	25	15	15	20	55	15	36
	HI-mild7	27	5	5	0	65	55	55	0	5	5	60	50	60	38	30
	HI-mild8	64	35	25	30	35	55	75	50	45	50	55	60	95	40	52
	HI-mild9	77	25	25	20	45	75	100	15	15	15	20	50	75	28	45
	HI-mild10	72	15	15	10	25	50	70	20	20	15	30	65	70	28	49
HI moderate																
	HI-mod1	71	10	10	25	50	55	50	25	20	25	45	60	25	43	48
	HI-mod2	65	45	35	30	35	60	55	50	45	30	35	65	45	42	40
	HI-mod3	58	35	35	45	70	55	80	30	40	50	60	55	80	55	55
	HI-mod4	67	30	25	30	50	65	75	30	35	55	65	75	85	48	62
	HI-mod5	63	20	20	60	75	80	80	20	15	55	75	70	65	67	53
	HI-mod6	57	20	25	35	75	85	100	20	25	35	75	85	110	40	50
	HI-mod7	72	25	20	25	50	55	95	20	20	25	45	55	100	42	48
	HI-mod8	72	35	35	30	55	55	60	50	50	55	55	60	75	47	67
	HI-mod9	81	35	35	35	45	60	75	35	35	40	50	70	80	47	58
	HI-mod10	74	25	30	45	50	50	85	20	25	40	40	65	70	48	53
Excluded participants																
	—	72	45	35	40	60	100	95	55	45	45	75	110	125	67	72
	—	77	55	55	55	60	95	90	55	50	55	55	90	120	67	83
